# Mismatches between UK food supply and dietary guidelines: a dietary gap assessment

**DOI:** 10.1017/S1368980025100633

**Published:** 2025-07-10

**Authors:** Niamh M. Kelly, Rebecca Wells, Rosalind Sharpe, Christian Reynolds

**Affiliations:** 1 Centre for Food Policy, City St George’s, University of London, London, UK; 2 Centre for Biodiversity and Environment Research, University College London, London, UK; 3 Centre for Agriculture, Food and Environmental Research, School of Health, Medicine and Life Sciences, University of Hertfordshire, Hatfield, UK

**Keywords:** Food systems, Agriculture, Healthy diets, Dietary recommendations, Food policy

## Abstract

**Objective:**

To examine how aligned the UK food supply is with the Eatwell Guide and identify discrepancies that should be addressed to support the availability of healthy diets for the population.

**Design:**

A dietary gap assessment was carried out on the 2022 UK food supply with FAOSTAT Food Balance Sheets (FAO-FBS) data, including domestic production, imports and exports and excluding animal feed, seeds and non-food uses. Foods were grouped into potatoes and cereals, oils and spreads, dairy products, protein, fruit and vegetables and sugar. The percentage contribution of each food group to the food supply was compared with the Eatwell Guide. An overview of the food supply from 2010 to 2022 was also created. To triangulate the data, FAO-FBS data were compared with the 2022 data from the Department for Environment, Food and Rural Affairs (DEFRA).

**Setting:**

UK, 2010–2022

**Participants:**

N/A

**Results:**

The proportion of fruit and vegetables, potatoes and cereals in the UK food supply was lower than the Eatwell Guide, while dairy products and oil were higher. Only 7 % of the food produced in the UK in 2022 was fruit and vegetables. This was the second smallest proportion, after oils and spreads (6 %), and about half the amount of sugar beet produced (13 %).

**Conclusion:**

Although the relationship between food supply and consumption is complex, taking a more coherent approach by integrating dietary recommendations with the food supply could help increase the availability of the recommended healthy diet. Going forward, DEFRA should include dietary gap assessments in future Food Security Reports.

The FAO states that food-based dietary guidelines are ‘intended to set out the dietary “vision” for the country and establish the basis for public food and nutrition, health and agricultural policies and nutrition education programmes’ (p. 11)^(1)^. However, many aspects of the food system and related policies are out of alignment with dietary guidelines in the United Kingdom (UK) represented in a food guide called the Eatwell Guide^(2)^. This can be seen through the lack of affordability, accessibility and availability of recommended diets^(3–5)^. Despite the promotion of the UK food-based dietary guidelines, compliance is very low^(6)^. A secondary analysis of several observational studies, including a total of 557 722 participants in the UK, found that less than 1 % of the study population met all nine recommendations in the Eatwell Guide, and only about 30 % met at least five recommendations^(6)^.

Incorporating a dietary guideline lens when assessing aspects of the food system could help to ensure the guidelines are operationalised as the vision, or goal, of food system activities and policies. While there are efforts to improve compliance with dietary guidelines, the focus is often based on education and promoting dietary change amongst the public^(7–9)^. Alignment of the food supply with the national recommended healthy diet could also contribute to the fulfilment of the right to food, which states that governments have a responsibility to ensure the whole population has access to adequate, healthy food^(10)^. The UK government ratified the UN’s International Covenant on Economic, Social and Cultural Rights, meaning the government is legally bound to ensuring the right to food for the population^(11)^. The availability of adequate food is defined as ‘the availability of food in a quantity and quality sufficient to satisfy the dietary needs of individuals, free from adverse substances, and acceptable within a given culture’ (p. 3)^(10)^. This study uses the Eatwell Guide as a framework to evaluate how well the UK food supply matches up with healthy dietary advice to support the availability of adequate, healthy food for all.

While there are health benefits to eating in line with dietary guidelines, there is also evidence to suggest that compared with current diets, recommended diets may offer many environmental benefits such as reduced greenhouse gas emissions and less land used for agriculture^(12)^. In the secondary analysis mentioned above, Scheelbeek and colleagues found that diets with high-to-moderate adherence to the Eatwell Guide had 30 % lower diet-related greenhouse gas emissions compared with those with very-low adherence^(6)^. Ensuring the availability of the recommended healthy diet, therefore, could help to promote both human and planetary health.

The dietary gap assessment is an emerging method to assess how aligned a food supply is with national dietary guidelines^(13–15)^. This approach, developed by Kuyper and colleagues, assesses the ‘degree to which a nation’s current food supply could meet the goal of achieving “healthy” diets’ (p. 2277)^(13)^. Kuyper *et al.* compared the food supply for Cameroon to the Dietary Approaches to Stop Hypertension (DASH) diet using 2011 data from FAOSTAT Food Balance Sheets (FAO-FBS). Authors assigned each food in the FAO dataset to the seven groups of the DASH diet, excluding sugar, alcohol, beverages, coffee and spices, which were not included in the recommended diet. Compared with the DASH diet, the food supply in Cameroon did not have enough animal-sourced foods or fruit and vegetables if tubers and plantain were categorised as starches instead of fruit and vegetables^(13)^. While the Eatwell Guide has been used as the basis for some dietary scenarios in the UK^(16)^, to the best of our knowledge, a full dietary gap assessment for the UK has not been published.

This study focuses on the availability of healthy diets, specifically looking at how the current UK food supply compares with the recommendations of the Eatwell Guide, and identifies areas where changes are needed. This research goes beyond the standard dietary gap assessment, also looking at how the breakdown of the food supply differs based on domestic production, imports and exports in 2022 and from 2010 to 2022. This assessment could help to inform national food strategies and food systems policies by identifying a baseline and highlighting where changes may be needed in the food supply to ensure the UK food system is supporting the availability of a healthy diet. To this end, we conclude with recommendations for policy and practice.

## Method

A dietary gap assessment was carried out to establish the composition of the UK food supply and identify what changes might be needed to better align the supply with the recommended healthy diet^(13)^. The Eatwell Guide, published in 2016 by Public Health England to help communicate the national dietary guidelines, was used as the healthy diet standard^(2)^. This food guide provides the percentage contributions each food group should make to the overall diet (in weight) and has a set of nine dietary recommendations^(17)^. The nine specific recommendations focus on the energy contribution of each macronutrient to the diet, sodium and fibre intake and serving recommendations for fruit and vegetables, fish and red and processed meat. While Scarborough and colleagues provide specific weights for each food group in their optimisation modelling to determine the proportions of each segment of the Eatwell Guide^(18)^, the current analysis focused on the information included in the dietary guideline documentation^(17)^. The percentage contribution of each food group to the weight of total dietary intake, provided in the Public Health England overview of the Eatwell Guide, was deemed the most appropriate guidance to use for this analysis as it included all food groups^(17)^.

**Fig. 1:**
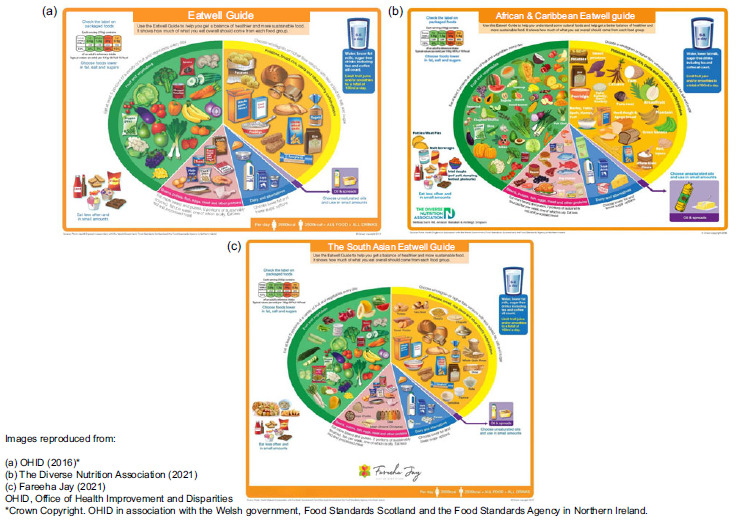
Eatwell Guide: (a) UK Government Publication, (b) African and Caribbean Eatwell Guide and (c) South Asian Eatwell Guide.

New iterations of the Eatwell Guide have been developed based on African and Caribbean and South Asian diets by The Diverse Nutrition Association^(19)^ and dietitian Fareeha Jay^(20)^, respectively. These versions of the guide present the same foods and food groups in the same proportions as the original Eatwell Guide, with the addition of foods commonly found in African and Caribbean or South Asian diets. The visuals for each version of the Eatwell Guide are shown in Fig. 1.

### Food supply data

To estimate the food supply, all UK food supply data for 2010–2022 were downloaded from FAOSTAT Food Balance Sheets (FAO-FBS)^(21)^ in 1000 tonnes and were converted to million tonnes. The food supply (*FS*) was calculated according to Equation 1. The separated data for UK production 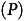 , imports 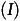 and exports 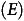 were assessed to understand how much of each food group consumed in the UK was produced within the country or imported. As these datasets include food for all forms of use, the total amount of food in each food group used for animal feed 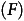 , seeds 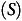 and ‘non-food uses’ 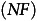 was subtracted from the total domestic food supply, production and imports. Data on food lost during processing were not factored into these calculations, as while the data are provided by FAO, this analysis was compared with data from the Department for Environment, Food and Rural Affairs (DEFRA) – discussed further at the end of this section – which does not include the same level of processing data for each food group. Figure A.1 in the Appendix shows the dietary gap assessment when food lost during processing is accounted for. The proportion of animal feed produced in the UK compared with imports was calculated to be 79 % and 21 %, respectively. This was derived from the Agriculture and Horticulture Development Board data on the supply of animal feed for 2021/2022 and 2022/2023^(22)^. As the proportion of food produced or imported for non-food uses was unclear, food for non-food uses was divided 50/50 between production and imports. It was assumed that all foods reserved for seed were from domestic production.
[1]

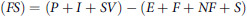




where *FS* is food supply, *P* is production, *I* is imports, *SV* is stock variation, *E* is exports, *F*is animal feed, *NF* is non-food uses and *S* is seed.

A total of ninety-four foods were available in the dataset. Foods that were included in the analysis (*n* 72) were grouped into the same categories as the Eatwell Guide: potatoes, bread, rice, pasta and other starchy carbohydrates (referred to hereafter as ‘potatoes and cereals’) (*n* 15); fruit and vegetables (*n* 17); beans, pulses, fish, eggs, meat and other proteins (referred to as ‘protein’) (*n* 20); dairy products (*n* 2); and oil and spreads (*n* 18)^(2,19,20)^. Tea, coffee, cocoa beans, cottonseed and cottonseed oil, alcohol, spices and infant food were removed, as they are not included in the food groups of the Eatwell Guide^(2)^.

The content of food groups in the Eatwell Guide, including the South Asian and African and Caribbean Eatwell Guides, was checked to categorise foods^(2,19,20)^. When there was any uncertainty about particular foods, which were not included in the graphics of the Eatwell Guides and did not obviously fit into a food group, the methodology of Stewart and colleagues was consulted as they categorised the same dataset to fit the EAT-Lancet diet^(23)^. The full list of foods included, and their respective food groups, can be found in Table B1 in Appendix B.

Sugar was included as a group for analysis despite not appearing directly on the plate in the Eatwell Guide as it contributed a sizable proportion of the food supply. When food items were being coded for sugar, a distinction was made between sugar beets – the crops used to produce sugar in the UK – and the ‘raw equivalent’ of refined sugar, along with sweeteners and honey (altogether coded as ‘sugar and sweeteners’). Sugar beet was coded as ‘sugar crops’, and this was used when calculating food production to identify the total weight of raw products. However, very little sugar beet is imported into the UK. Based on data from FAO-FBS, imported sugar is mostly in the form of the ‘raw equivalent’ of sugar, rather than sugar beets. Therefore, the ‘raw equivalent’ of sugar was used to calculate the overall contribution of sugar (along with honey and sweeteners) in the food supply, imports and exports as this is closer to what is consumed by the public and is mostly traded in this form. The value for raw sugar cane was zero for all production, imports and exports and was therefore excluded from analysis.

All foods were categorised into their respective groups from the Eatwell Guide or excluded as mentioned above, using RStudio statistical software^(24)^, and the total weight of each food group for each year was obtained. The results were downloaded to Excel, and Datawrapper was used to create graphs^(25,26)^. The percentage contribution of each food group to the supply (in terms of weight) was compared with the percentage contribution recommended in the Eatwell Guide. If a food group contributed a higher or lower percentage to the food supply compared with the recommendation in the Eatwell Guide, the food group was categorised as ‘too high’ or ‘too low’, respectively. Percentage contribution by weight was used instead of calories, which was used by other studies^(13)^, as that is how the Eatwell Guide is presented^(2)^.

### Comparison of FAO-FBS and DEFRA data

The overall breakdown of the food supply was compared between the FAO-FBS data^(21)^ and data from DEFRA^(27)^ to assess the validity of using FAO-FBS data for the dietary gap assessment. The breakdown of the food supply created from FAO-FBS was compared with DEFRA’s ‘Agriculture in the UK’ dataset for 2022 (chapters 7, 8 and 13)^(27)^. As with the data from FAO-FBS, individual foods were grouped based on the Eatwell Guide (Table B2, Appendix B). The ‘total new supply’ for 2022 was extracted for each food that was available. The total weight of potatoes and cereals reported for animal feed and seeds was subtracted from the ‘total new supply’ of potatoes and cereals. The 2022 report on Fisheries in the UK was used to quantify the supply of fish during that period^(28)^.

As with the FAO-FBS dataset, food groups in the DEFRA dataset were categorised as ‘too high/low’ or on target in comparison with the recommended proportions in the Eatwell Guide. The results were compared with the FAO-FBS dietary gap assessment to assess whether the two datasets aligned on the discrepancies identified between the food supply and the Eatwell Guide. The quantities of each food group being produced in the UK, imported and exported were also compared between the datasets.

## Results

**Fig. 2:**
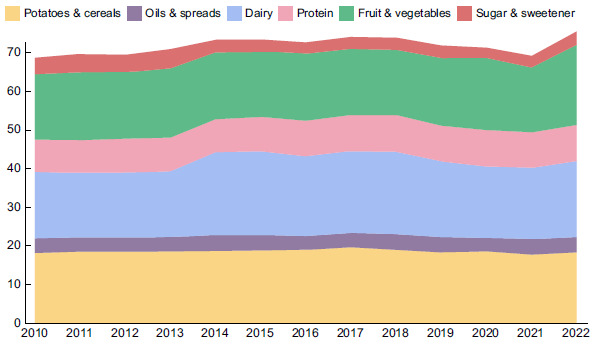
UK Domestic food supply 2010–2022 (million tonnes). Includes foods produced domestically and imported, and excludes exports, as well as foods for animal feed, seeds and non-food uses.

First, the composition of the UK food supply was assessed from 2010 to 2022 using FAO-FBS data to identify any trends (Fig. 2). As the food system and particularly agricultural production and trade were impacted during the pandemic in 2020/21, it was important to check whether the most recent data are representative of current trends. However, the overall composition of the food supply remained relatively stable since 2010, with an upward trend in the total amount of food, increasing from 68·76 to 75·53 million tonnes from 2010 to 2022. Food production in the UK and exports also remained relatively stable over the long term (45·42 *v*. 47·71 million tonnes of food production and 14·41 *v*. 13·18 million tonnes of exports in 2010 and 2022, respectively). The main increase in the food supply comes from imported food, which has increased from 39·45 to 49·44 million tonnes from 2010 to 2022. The increase predominantly came from imported fruit and vegetables, which increased from 14·46 to 19·59 million tonnes from 2010 to 2022. In the same period, UK production of fruit and vegetables declined slightly (3·20–3·11 million tonnes, respectively), with only minor fluctuations from year to year.

When looking at the breakdown of the food supply during the COVID-19 pandemic, it does not seem that the overall weight of total food or the composition of the UK food supply was significantly impacted. However, when looking more closely at domestic production, imports and exports, there are variations across 2020/2021. The quantity of food production dipped from 51·80 million tonnes in 2019 to 41·95 million tonnes in 2020. However, this was counteracted by increased imports from 43·87 million tonnes to 46·09 million tonnes and a reduction in exports from 15·05 to 13·81 million tonnes in 2019 and 2020, respectively. These changes, combined with changes in stocks (stores) of food, meant the total quantity of food in the supply remained mostly stable. There were also some differences observed from 2020 to 2021, when imports reduced from 46·09 million tonnes in 2020 to 40·13 million tonnes in 2021, and exports reduced again to 10·68 million tonnes in 2021. In both cases, the reduction in imports was partially offset by the reduction in imports. The figures showing these breakdowns in more detail can be found in Appendix D.

### Dietary gap assessment

**Fig. 3:**
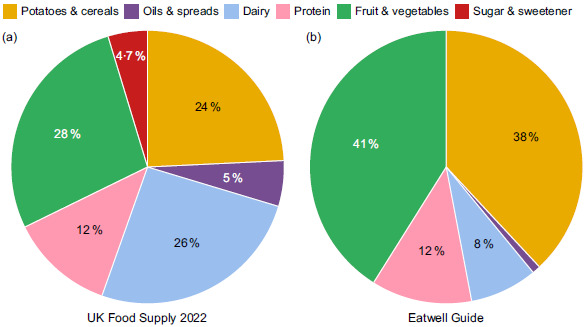
Dietary Gap Assessment UK 2022: (a) UK food supply based on FAOSTAT FBS data, (b) Eatwell Guide. Excludes foods used for animal feed, seeds and non-food uses.

The UK supply of food for human consumption in 2022 (Fig. 3(a)) has a higher proportion of dairy products, oils and spreads, and sugar and a much lower proportion of fruit and vegetables and potatoes and cereals than recommended in the Eatwell Guide (Fig. 3(b)). While sugar is not represented in a segment within the Eatwell Guide, it was included in Fig. 3 to highlight the proportion it contributed to the food supply. A version of the pie chart without sugar can be seen in Appendix C. The proportions of other food groups are relatively similar with and without sugar being included in the breakdown, particularly when considering if there was too much or too little of a food group. The biggest discrepancies were fruit and vegetables and potatoes and cereals. Fruit and vegetables represent approximately 41 % of the diet in the Eatwell Guide, but only 28 % in the 2022 UK food supply, when sugar was included. The proportion of protein was the same in both the food supply and recommendations.

### Animal feed

While the dietary gap assessment presented above only includes food for human consumption, the discrepancies identified are more pronounced when animal feed is included in the analysis. When looking at the total food and animal feed supply, approximately 41 % of all potatoes and cereals produced and imported in the UK were used for animal feed in 2022 (15·09 million tonnes). If the total food and feed supply is used for a dietary gap assessment, and foods for non-food uses and seeds are removed, potatoes and cereals represent 36 % of the supply, compared with 24 % when animal feed is removed. Meanwhile, fruit and vegetables represent 22 % of the food and feed supply. Figures E.1 and E.2 in the online Supplementary material show the dietary gap assessment and the breakdown of the food and feed supply by domestic production, imports and exports with animal feed included.

### The origins of UK food

**Fig. 4:**
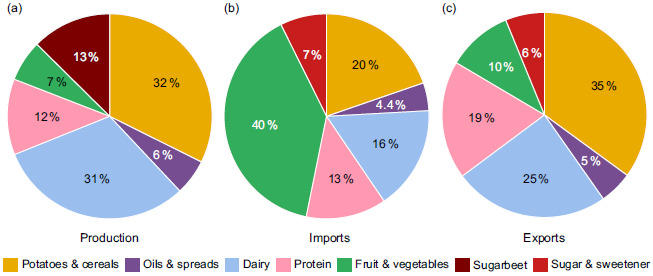
UK 2022 food supply breakdown by (a) production in the UK, (b) imports, (c) exports. Excludes foods used for animal feed, seeds and non-food uses.

The food supply was broken down into domestic production, imports and exports (Fig. 4). While the UK produces approximately 60 % of food domestically, this varies based on food groups, with potatoes and cereals (85 %) and dairy products (75 %) having the highest self-sufficiency, based on FAO-FBS data. As mentioned at the beginning of the results section, the majority of the increase in the UK food supply has come from increased imports, particularly fruit and vegetables. Fruit and vegetables have the lowest self-sufficiency rate, with only about 15 % of fruit and vegetables in the 2022 UK food supply being produced within the UK, according to FAO-FBS data. In the same year, after removing the food used for animal feed, seed and non-food uses, only about 7 % of the food produced in the UK was fruit and vegetables. At the same time, 13 % of UK food production was sugar beet.

### Comparison of UK food supply using FAO-FBS and DEFRA datasets

**Fig. 5:**
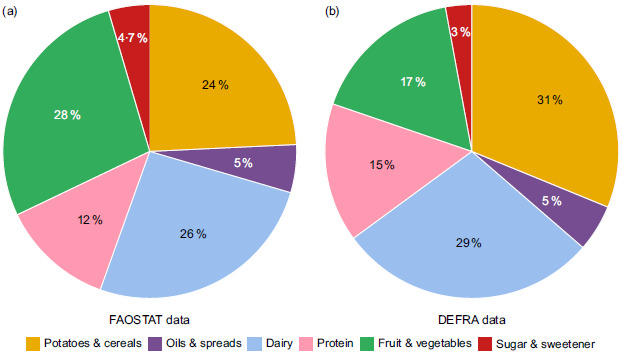
Comparison of UK food supply with (a) FAOSTAT FBS and (b) DEFRA data. Excludes foods used for animal feed, seeds and non-food uses.

The breakdown of the overall food supply is similar between the FAO-FBS data and DEFRA statistics, in that they both identify that the proportion of the food supply composed of fruit and vegetables and potatoes and cereals are too low and the proportions of dairy products and oil are too high (Fig. 5). However, the proportion of the supply made up of potatoes and cereals is much higher in the DEFRA data at 31 % of food supply, compared with the FAO-FBS results of 24 %. On the other hand, fruit and vegetables form a smaller proportion of the food supply based on DEFRA data (17 % *v*. 28 % from FAO-FBS). When comparing the actual weight of each food group, rather than the percentage contribution to the food supply, the similarities are more mixed. Despite potatoes and cereals representing a larger proportion of the overall food supply based on DEFRA data, the total weight of potatoes and cereals in the food supply is actually lower when calculated from DEFRA’s dataset (15·09 million tonnes) compared with FAO-FBS’s (18·31 million tonnes). Table 1 shows a comparison of the quantity in each food group in million tonnes for both datasets.

**Table 1.** The UK food supply in 2022 based on data from FAOSTAT FBS and DEFRA in million tonnes

	FAOSTAT				DEFRA			
Food group	Total supply	Production	Imports	Exports	Total supply	Production	Imports	Exports
Potatoes and cereals	18·31	15·43	9·74	4·61	15·09	14·75	2·71	2·43
Oils and spreads	4·07	2·72	2·19	0·69	2·55	1·36	0·81	0·05
Dairy products	19·56	14·77	8·08	3·2	13·83	14·95	0·18	0·89
Protein	9·27	5·67	6·26	2·46	7·77	6·42	2·42	1·06
Fruit and vegetables	20·77	3·11	19·59	1·37	8·20	3·01	5·32	0·13
Sugar and sweetener	3·56	0·97	3·57	0·8	1·376^*^	0·75^*^	0·67^*^	0·06

DEFRA, Department for Environment, Farming and Rural Affairs; FBS, Food Balance Sheets.

Food for Animal feed, seeds and non-food uses have been removed. Values for stock variation are not included in this table.

* No value available for sweetener.

Discrepancies between the datasets are particularly apparent when comparing imports. The data on the production of fruit and vegetables in the UK in 2022 are quite similar between both datasets: according to DEFRA statistics, 3·01 million tonnes of fruit and vegetables were produced in the UK (including fresh vegetables, fresh fruit and field peas), while based on the FAO-FBS, 3·11 million tonnes were produced. However, the import quantity of fruit and vegetables from FAO-FBS (19·59 million tonnes) is nearly four times larger than what is reported by DEFRA (5·32 million tonnes). This leads to varying rates of self-sufficiency for fruit and vegetables, where, according to DEFRA data, 40 % of fruit and vegetables were produced in the UK in 2022 (3·01/8·2 million tonnes), in contrast to 15 % based on FAO-FBS data.

Similarly, values on the production of potatoes and cereals are relatively comparable between the two databases. DEFRA reported 14·75 million tonnes of potatoes and cereals produced (excluding animal feed and non-food uses), while FAO-FBS reports 15·43 million tonnes produced in the UK in 2022. However, the total weight of imported potatoes and cereals varies more, with FAO-FBS reporting 9·74 million tonnes imported, compared with 2·72 million tonnes of imported cereals and potatoes based on DEFRA data.

## Discussion

There are fundamental disparities between the UK food supply and the Eatwell Guide, with a lower proportion of potatoes and cereals for human consumption and fruit and vegetables in the supply compared with recommendations and too much sugar, dairy products and oils and spreads. These discrepancies need to be addressed as they can contribute to systemic barriers to healthy eating, impacting the availability of healthy diets for the population. Additionally, this analysis further highlighted the UK’s reliance on imported food, particularly fruit and vegetables. The overall composition of the food supply has been stable over the last decade. Interestingly, while the composition of the overall food supply did not seem to be substantially impacted during the COVID-19 pandemic in 2020/21 or ‘Brexit’ – withdrawal of the UK from the European Union – which came into effect after January 2020^(29)^, there are noticeable decreases in UK food production and imports in 2020/21, which may have been offset by the reduction in exports during that period.

The results of this dietary gap assessment are in line with previous studies on both a national and a global scale: the food supply commonly does not match up with recommended healthy diets. Research by Bahadur and colleagues compared the global food supply with the Harvard Healthy Eating Plate (HHEP)^(14)^. Using data from the 2011 Global FAO-FBS, foods were organised into wholegrains, fruit and vegetables, protein, milk and oils, based on the five groups in the HHEP. As in the current analysis, the authors found the global food supply contained too many fats and sugars, but not enough fruit and vegetables. However, in contrast to the present study, Bahadur and colleagues found the global food supply had too many grains and not enough protein compared with the HHEP^(14)^. This lack of protein is likely due to the lower supply of protein, particularly animal-sourced protein, in low- and middle-income countries, while often high-income countries have enough, or sometimes too much, protein in the food supply^(30)^.

A report by the Food Research Collaboration compared data on UK household purchasing habits in 2013 from DEFRA Food Statistics Pocketbook with the Eatwell Guide and found similar results^(31)^. Households were not purchasing enough fruit and vegetables to meet recommendations while purchasing too many dairy products and high-fat and sugar products and a similar amount of meat as is recommended^(31)^. As in the present study, the analysis of purchasing habits found lower purchasing of potatoes and cereals than is recommended. This report also highlighted poor access and availability of fruit and vegetables as one of the barriers to consumption^(31)^.

When comparing the UK food supply to the EAT-Lancet diet, rather than the Eatwell Guide, the results are similar^(23)^. Stewart *et al.* used the same FAO-FBS data set to compare the UK food supply with the EAT-Lancet diet, and although the food groups were slightly different to the current analysis, the results agree on an excess of beef, lamb and pork and dairy products and too little vegetables, nuts and legumes^(23)^.

In addition to the overall lack of fruit and vegetables in the UK food supply, the majority of the fruit and vegetables in 2022 were imported. According to FAO-FBS data, only 15 % of fruit and vegetables were produced domestically that year, and only 7 % of food produced in the UK in 2022 was fruit and vegetables. The reliance on imported fruit and vegetables increased between 2010 and 2022 (from 14·46 to 19·59 million tonnes). This trend was also found by de Ruiter and colleagues when assessing the food supply from 1986 to 2009^(32)^. Similarly, DEFRA reported an increase of 13 % in imported fruit and vegetables from 2003 to 2023^(33)^. In the 2024 UK Food Security Report, DEFRA highlighted the risk brought about by climate change and the dependence on a small number of countries for the security of the fruit and vegetable supply in the UK^(33)^. The over-reliance on imports for fruit and vegetables is a risk to the resilience of the supply, particularly because about one-third of fruit and vegetables imported to the UK are from countries vulnerable to climate change^(34)^. The impacts of this were clearly seen in Spring 2023, also featured in the 2024 Food Security Report^(33)^, when the UK experienced shortages of tomatoes as major producer countries were affected by changes in weather patterns at the same time UK producers faced prohibitively high energy costs for controlled environment horticulture, such as greenhouses that are used to elongate the growing season^(33,35,36)^. With UK agriculture also being strongly affected by changing weather patterns in recent years^(33)^, amongst other barriers to production, the lack of domestically produced fruit and vegetables could be exacerbated without support from the government to increase production and improve the resilience of the sector.

Any shift in the food supply towards the dietary guidelines would also need to consider economic impacts on farmers. Arnoult and colleagues carried out a modelling study of the regional impacts of shifting food consumption to the previous version of the Eatwell Guide in England and Wales, using dietary data from the Expenditure and Food Survey 2003–2004^(37)^. Alongside changes to the diet, they estimated that the net profit in the two nations would rise, but some regions, mostly those best suited to livestock, would be negatively affected as they could not benefit from an increased demand for cereals and horticulture^(37)^. This highlights the need to consider the impacts on the livelihoods of different farmers when these transitions are made.

The environmental impacts of this mismatch should also be considered. This analysis highlighted that the UK grows almost twice as much sugar beet as fruit and vegetables. This is concerning as not only are there major differences between the impact of these foods on human health, but there are also concerns about the environmental impacts of sugar production^(38–40)^. Civil Society Organisations (CSOs) have highlighted the environmental damage linked with sugar beet production, particularly in the UK, as it removes substantial amounts of topsoil on some of the best quality land in the country, particularly East Anglia^(38,40)^. Sugar beet farming also heavily relies on neonicotinoid pesticides, which are harmful to pollinators^(38,40)^, and although these pesticides are currently not authorised, emergency authorisations have been granted for the last 4 years in England^(41,42)^. There have also been questions raised about whether healthier foods could be grown in these areas of high-quality land^(39)^. Moving the food supply closer to the dietary guidelines may also make room for more sustainable practices. The modelling study by Arnoult in England and Wales suggested that the reduction of livestock needed for a healthy diet would allow more space for grass-based systems, thereby helping to improve both human health and environmental sustainability^(37)^.

FAO-FBS is the standard dataset for dietary gap assessments^(13)^. These data were compared with the National ‘Agriculture in the UK’ dataset to triangulate the findings^(27)^. This comparison identified that while the proportions each food group contributed to the food supply were slightly different between datasets, both agreed on whether food groups fell into higher or lower proportions than recommended by the Eatwell Guide, except for proteins, which were slightly too high based on the DEFRA dataset. However, in terms of the actual quantity of each food group in the food supply, the FAO-FBS had a higher weight for each food group compared with DEFRA, particularly when it came to imports.

DEFRA and the FAO have different processes for collecting data on the food supply in the UK. In terms of trade data, the FAO sources UK trade information from the UN trade database ComTrade^(43)^, while DEFRA data are compiled from transaction information collected by His Majesty’s Revenue and Customs (HMRC). As production levels are relatively similar between databases, the main discrepancies seem to stem from data on imports. There are also differences in the foods included in different categories, for example, Agriculture in the UK Trade dataset (Chapter 13) states in the notes that ‘fresh fruit and vegetables’ do not include jams, juice, dried or processed fruit, dried legumes or processed vegetables^(27)^. Meanwhile, the FAO-FBS dataset includes dried, canned and processed fruit and vegetables, as well as dried peas^(21)^.

In a comparison of UK FAO-FBS with DEFRA’s Household Budget Surveys (HBS), Smith and colleagues also found that FAO-FBS reported higher quantities of all food groups compared with the DEFRA-HBS^(44)^. As the HBS was based on foods purchased in the UK, this accounts for retail waste, unlike the FAO Food Balance Sheet, which only reports food supply. However, since the present study was comparing two food supply datasets, and therefore neither has been adjusted for food waste, this would not explain the higher levels of foods reported by the FAO data. Interestingly, Smith *et al*. did not include cereals or fruit and vegetables as a category in their analysis, as mentioned in their results, due to differences in how categories were defined and what form foods were reported in (e.g. bread in HBS and wheat in FAO-FBS). Smith and colleagues recommend using both FAO and DEFRA datasets in parallel when possible to monitor dietary trends^(44)^, as was the case in the present study.

### Policy and practice implications

Now that these discrepancies between the UK food supply and the recommended healthy diet have been identified, there needs to be policy support to bridge these gaps. As the UK is still in the early stages of post-Brexit policy change and has had a change of government in the past year (July 2024), there is potential to use this as an opportunity to align national policies towards shared goals, or at least, prevent contradictions. When developing agriculture and trade policies, the UK government should consider how these policies may affect the availability of the recommended healthy diet. More policy support in particular should be given to horticulture production within the UK to help the sector grow and become more resilient and reduce the UK’s dependence on imports from climate-vulnerable countries^(34)^. Any policy changes would also need to consider the impacts these changes could have on farmers and ensure they are supported through the transition.

Development of future food strategies across the UK and the upcoming Land Use Framework^(45)^ should include considerations about how the food supply aligns with the Eatwell Guide. Additionally, the UK government should include a dietary gap assessment in the Food Security Report, which it has committed to publishing every 3 years^(33)^. While the Food Security Report does compare the quantity of some food groups to the Eatwell Guide, a dietary gap assessment could provide an overall picture of the alignment of the whole food supply with recommendations, highlight discrepancies and monitor progress towards the availability of healthy diets for all.

In recent years, there has been growing interest in applying dietary guidelines to the wider food system, rather than focusing on behaviour change^(46–49)^. FAO is developing guidelines for countries to create food systems-based dietary guidelines^(49)^. As mentioned in the Introduction, this approach to implementing dietary guidelines could contribute to a nation fulfilling the right to food for the population. Dietary gap assessments could be included in this process as a useful starting point for countries to establish what changes are needed in their food supply and potentially elsewhere in their food system to better support healthy diets. A multidisciplinary working group took this approach in the development of Ethiopian dietary guidelines in 2022^(15)^.

Nutrition researchers and professionals should be aware of the current mismatch between dietary guidelines and the food supply, in particular domestic production. To reduce this gap, we make the following suggestions. (1) Nutrition teams in supermarkets and other supply chain actors should monitor their sales (and the origin of their products) in relation to dietary guidelines to help them target the availability of healthier food options. (2) In addition to public health campaigns to increase alignment with the Eatwell Guide, nutrition professionals should further engage with agronomists, farmers and food producers to highlight the importance of aligning their production with dietary guidelines: interdisciplinary working will be key to achieving the availability of healthy diets for all. (3) Nutrition professionals within CSOs and industry groups should also advocate for policies that align food production with dietary guidelines to ensure a healthier food supply.

### Future research

This research raises questions for future researchers. First, how can the changes recommended above be brought about? This would need to be considered in terms of policy change, structural barriers for farmers, supply chain logistics and consumer demand. There also needs to be an increased focus on the impact of trade to ensure that any changes in practice by UK growers are not undermined or offset by an increase in imports. To match a potential change in supply, there should be consideration of how to support greater consumption of healthy foods, particularly for those who are currently unable to afford a healthy diet. Additionally, as the UK has made commitments to reach environmental targets, particularly for reducing emissions and improving biodiversity^(50)^, it would be useful to carry out research on how aligning the food supply in the UK with the recommended healthy diet might interact with efforts to meet environmental targets within the country.

### Strengths and limitations

The main strength of using the FAO-FBS data is that it is freely available for most countries worldwide and therefore means this method can be easily replicated by other countries. The food supply was calculated to take into account production, imports and exports, as well as animal feed, seeds and food for non-food uses. The results from FAO-FBS data were also triangulated with official UK government statistics to validate the categorisation of food groups as too high/low in the food supply.

Limitations of this approach include the focus on the overall food supply, not individual consumption, which does vary from the supply. Additionally, it does not account for food waste. Other limitations highlighted by Kuyper and colleagues are that it does not allow for seasonal or geographic analysis across country regions, and it is not possible to analyse by individual characteristics such as gender and socio-economic status^(13)^. However, as the aim of this study was to assess the alignment of the overall food supply in the UK, in terms of food that is produced and imported and removing exports, it was not appropriate to factor in food waste or regional or demographic differences.

### Conclusions

The dietary guidelines provide a helpful framework that can be used to support the sufficient availability of adequate, nutritious food for everyone. This study highlighted the discrepancies between the UK food supply and the Eatwell Guide, showing a lack of fruit and vegetables and an excess of dairy products, oils and spreads and sugar. While there was also a lower proportion of potatoes and cereals in the food supply for human consumption compared with recommendations, a large portion of potatoes and cereals produced and imported into the UK are going towards animal feed (41 %). A reduction in animal feed, for example, through a reduction in dairy cows and a reallocation of some potatoes and cereals to human consumption, could help address this mismatch. Furthermore, the breakdown of the food supply based on production, imports and exports highlighted the lack of domestic production of fruit and vegetables in particular. The development of future food strategies and agriculture and trade policies should include assessments of these mismatches and endeavour to minimise them. Specifically, more support is needed to increase domestic horticulture to tackle the insufficient supply.

## Supporting information

Kelly et al. supplementary material 1 Kelly et al. supplementary material

Kelly et al. supplementary material 2 Kelly et al. supplementary material

## Supplementary material

For supplementary material accompanying this paper, visit https://doi.org/10.1017/S1368980025100633.
