# Supplementary figures and images for: Mismatches between UK food supply and dietary guidelines: a dietary gap assessment
Source: Public Health Nutr. 2025 Jul 10;28(1):e121. doi: 10.1017/S1368980025100633 (PMC12465069; doi:10.1017/S1368980025100633)

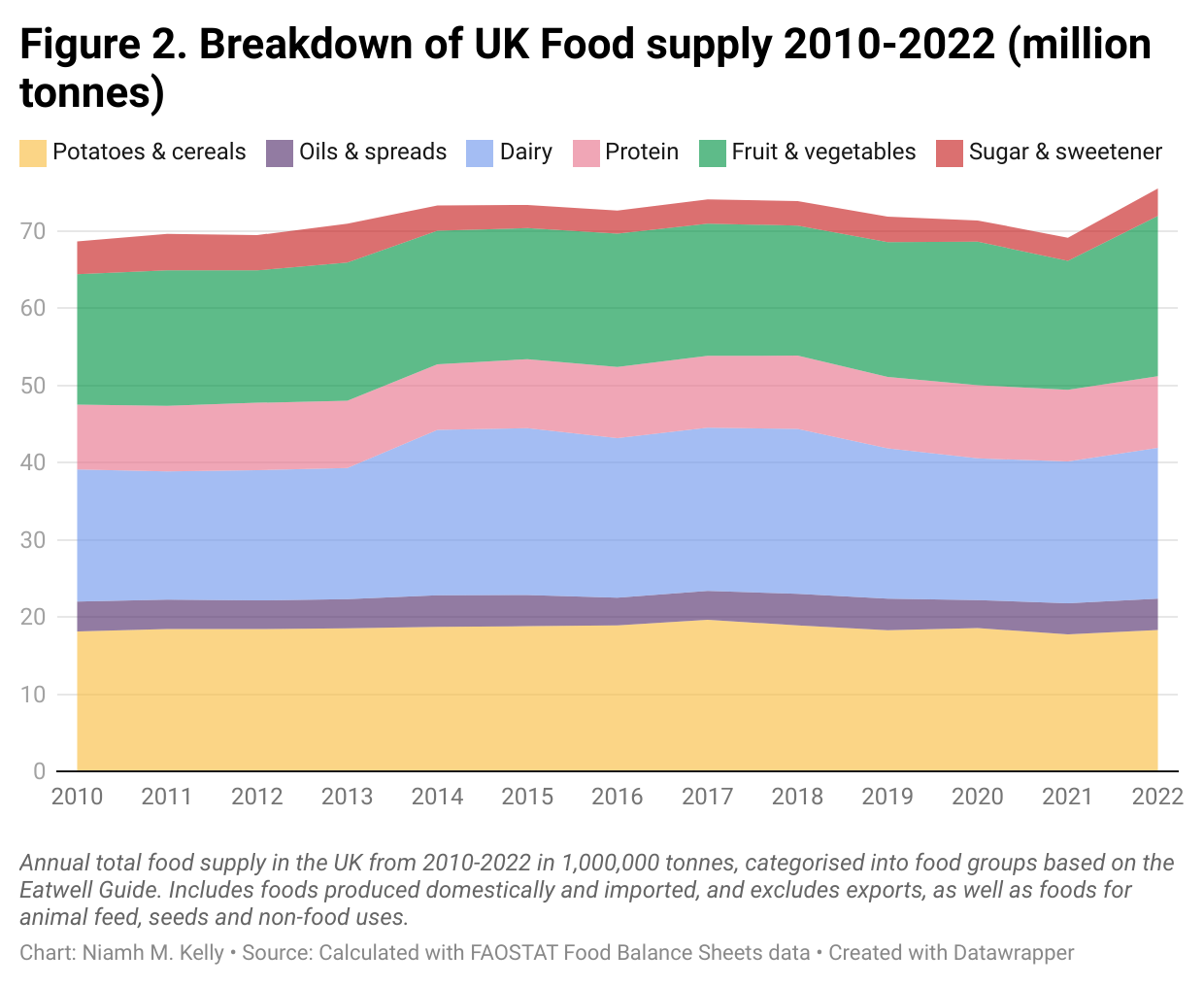

Supplement: Kelly et al. supplementary material 1 — Kelly et al. supplementary material [file S1368980025100633sup001.zip › Figure 2. UK Food Supply 2010-2022 RESUBMISSION.png]
