# Supplementary material for: Mismatches between UK food supply and dietary guidelines: a dietary gap assessment
Source: Public Health Nutr. 2025 Jul 10;28(1):e121. doi: 10.1017/S1368980025100633 (PMC12465069; doi:10.1017/S1368980025100633)

**Mismatches between UK food supply and dietary guidelines: A dietary gap assessment**

Niamh M. Kelly, Rebecca Wells, Rosalind Sharpe,^3^ Christian Reynolds

**Appendices**

**Appendix A**

**Figure A.1 UK Food supply 2022 (taking into account food lost during processing)**


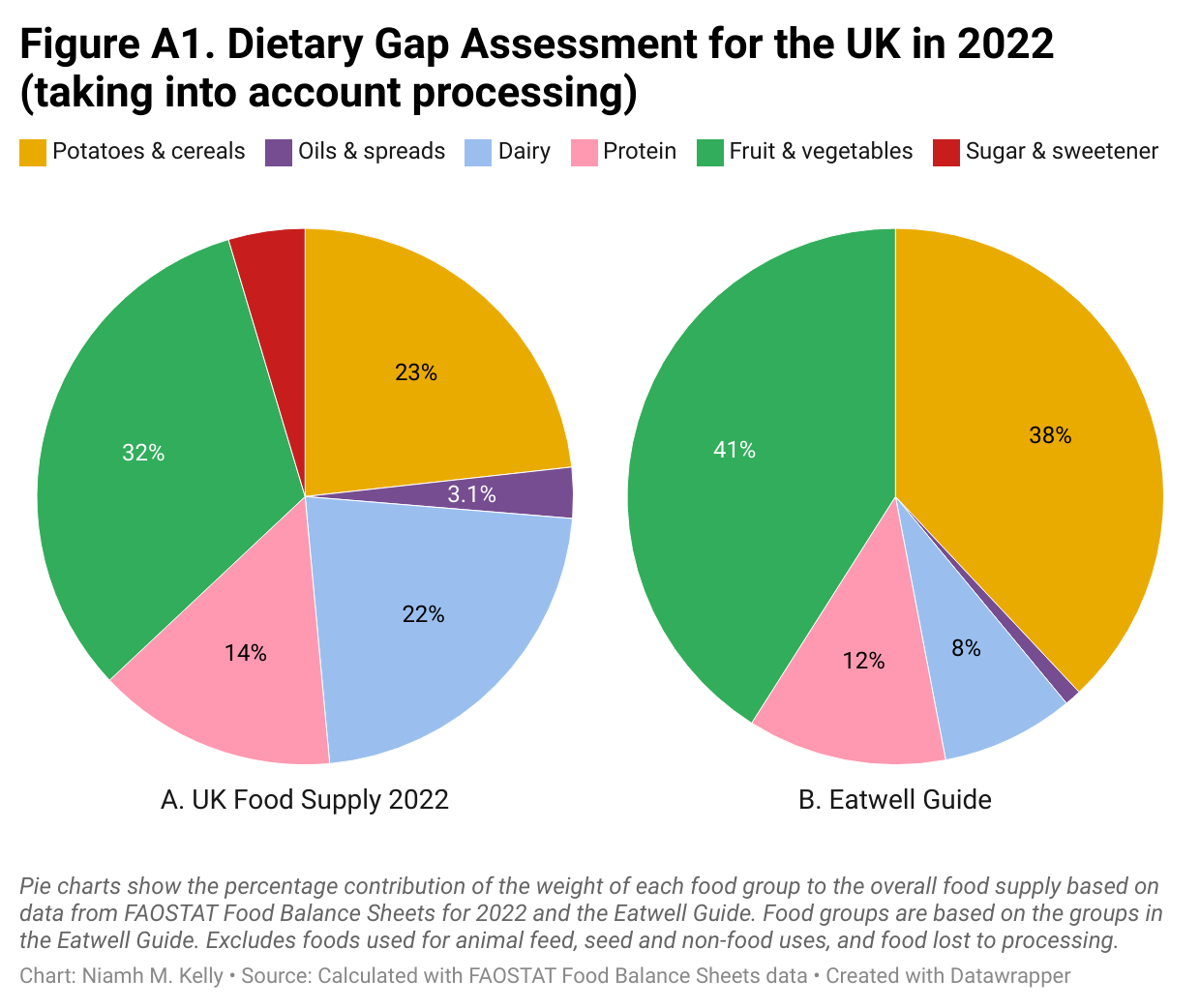


**Appendix B - Categorisation of foods in FAOSTAT Food Balance Sheets and DEFRA Agriculture in the UK dataset into Eatwell Guide food groups**

Food item listed as shown in FAOSTAT Food Balance Sheets (Table B1) and DEFRA Agriculture in the UK Statistics Chapters 7 and 8 (Table B2), and their corresponding Eatwell Guide food group.

.

**Table B.1. List of foods from FAOSTAT Food Balance Sheets, and their respective food groups based on the Eatwell Guide**

| Item | Food group |
| --- | --- |
| Apples and products | Fruit |
| Aquatic Animals, Others | Fish |
| Bananas | Fruit |
| Barley and products | Potatoes and cereals |
| Beans | Pulses |
| Bovine Meat | Red Meat |
| Butter, Ghee | Oils and spreads |
| Cassava and products | Potatoes and cereals |
| Cephalopods | Fish |
| Cereals, Other | Potatoes and cereals |
| Citrus, Other | Fruit |
| Coconut Oil | Oils and spreads |
| Coconuts - Incl Copra | Fruit |
| Cream | Dairy |
| Crustaceans | Fish |
| Dates | Fruit |
| Demersal Fish | Fish |
| Eggs | Protein |
| Fats, Animals, Raw | Oils and spreads |
| Fish, Body Oil | Oils and spreads |
| Fish, Liver Oil | Oils and spreads |
| Freshwater Fish | Fish |
| Fruits, Other | Fruit |
| Grapefruit and products | Fruit |
| Grapes and products (excl wine) | Fruit |
| Groundnut Oil | Oils and spreads |
| Groundnuts | Nuts |
| Honey | Sugar |
| Lemons, Limes and products | Fruit |
| Maize and products | Potatoes and cereals |
| Maize Germ Oil | Oils and spreads |
| Marine Fish, Other | Fish |
| Meat, Other | Meat |
| Milk - Excluding Butter | Dairy |
| Millet and products | Potatoes and cereals |
| Molluscs, Other | Fish |
| Mutton & Goat Meat | Red Meat |
| Nuts and products | Nuts |
| Oats | Potatoes and cereals |
| Offals, Edible | Meat |
| Oilcrops Oil, Other | Oils and spreads |
| Oilcrops, Other | Oils and spreads |
| Olive Oil | Oils and spreads |
| Olives (including preserved) | Fruit |
| Onions | Vegetables |
| Oranges, Mandarines | Fruit |
| Palm kernels | Oils and spreads |
| Palm Oil | Oils and spreads |
| Palmkernel Oil | Oils and spreads |
| Peas | Vegetables |
| Pelagic Fish | Fish |
| Pigmeat | Red Meat |
| Pimento | Vegetables |
| Pineapples and products | Fruit |
| Plantains | Potatoes and cereals |
| Potatoes and products | Potatoes and cereals |
| Poultry Meat | Meat |
| Pulses, Other and products | Pulses |
| Rape and Mustard Oil | Oils and spreads |
| Rape and Mustardseed | Oils and spreads |
| Rice and products | Potatoes and cereals |
| Roots, Other | Potatoes and cereals |
| Rye and products | Potatoes and cereals |
| Sesame seed | Seeds |
| Sesameseed Oil | Oils and spreads |
| Sorghum and products | Potatoes and cereals |
| Soyabean Oil | Oils and spreads |
| Soyabeans | Pulses |
| Sugar (Raw Equivalent) | Sugar |
| Sugar beet | Sugarbeet |
| Sunflower seed | Seeds |
| Sunflowerseed Oil | Oils and spreads |
| Sweet potatoes | Potatoes and cereals |
| Sweeteners, Other | Sugar |
| Tomatoes and products | Fruit |
| Vegetables, Other | Vegetables |
| Wheat and products | Potatoes and cereals |
| Yams | Potatoes and cereals |

**Table B.2 List of Foods in the DEFRA dataset, and their respective food groups based on the Eatwell Guide**

| Food item | Food Group |
| --- | --- |
| Beef | Protein |
| Butter | Oils and spreads |
| Cereals (wheat, barley oats) | Potatoes and cereals |
| Dairy | Dairy |
| Eggs | Protein |
| Fruit | Fruit and vegetables |
| Oilseed rape | Oils and spreads |
| Pigmeat | Protein |
| Potatoes | Potatoes and cereals |
| Poultry | Protein |
| Production of field beans | Protein |
| Production of peas | Fruit and vegetables |
| Sheep | Protein |
| Sugar (raw equivalent) | Sugar |
| Vegetables | Fruit and vegetables |

**Appendix C – UK Food Supply 2022 (excluding sugar)**

**Figure C.1 FAO-FBS UK Food supply for 2022 (excluding Sugar)**


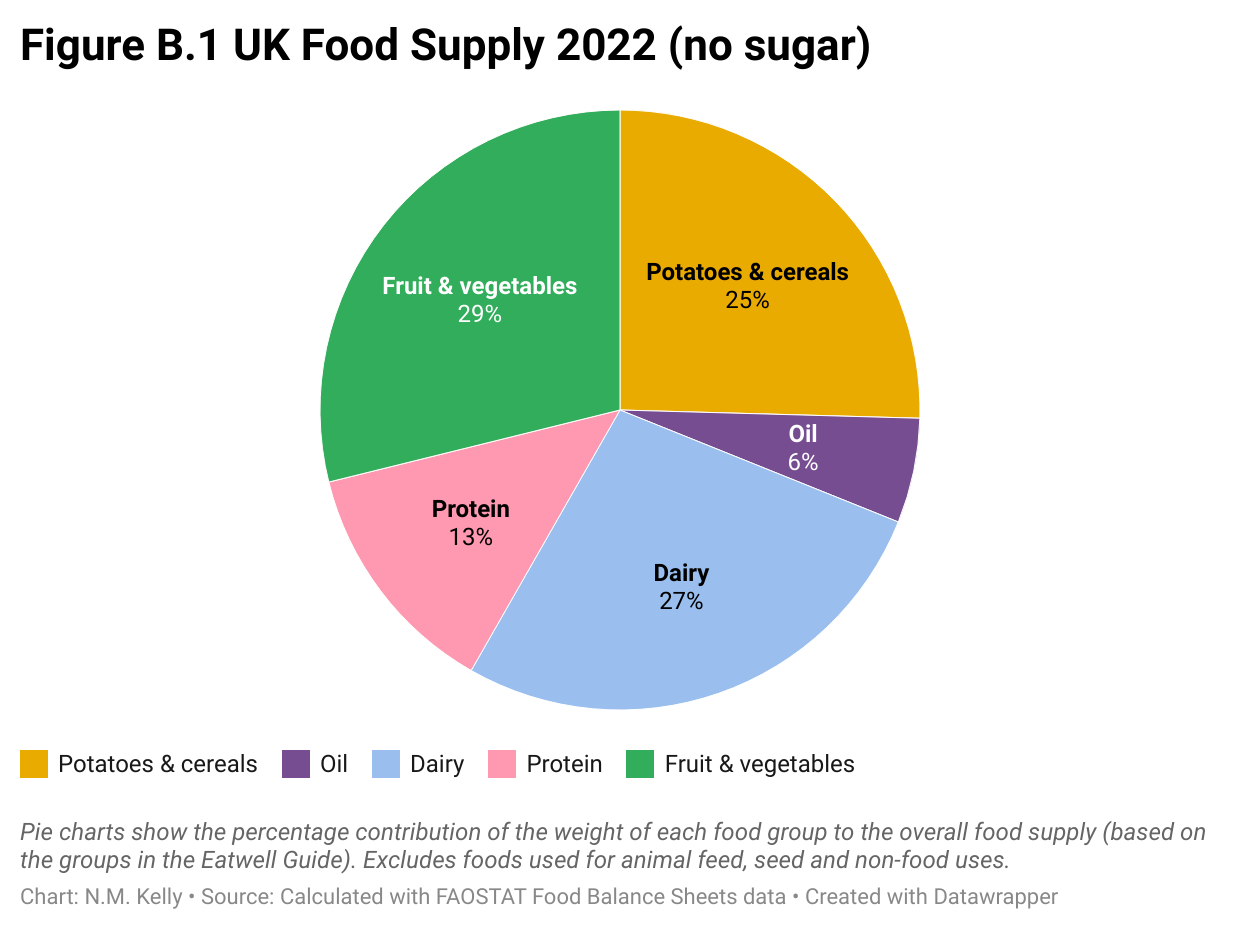


**Figure C.2 DEFRA UK Food supply for 2022 (excluding sugar)**


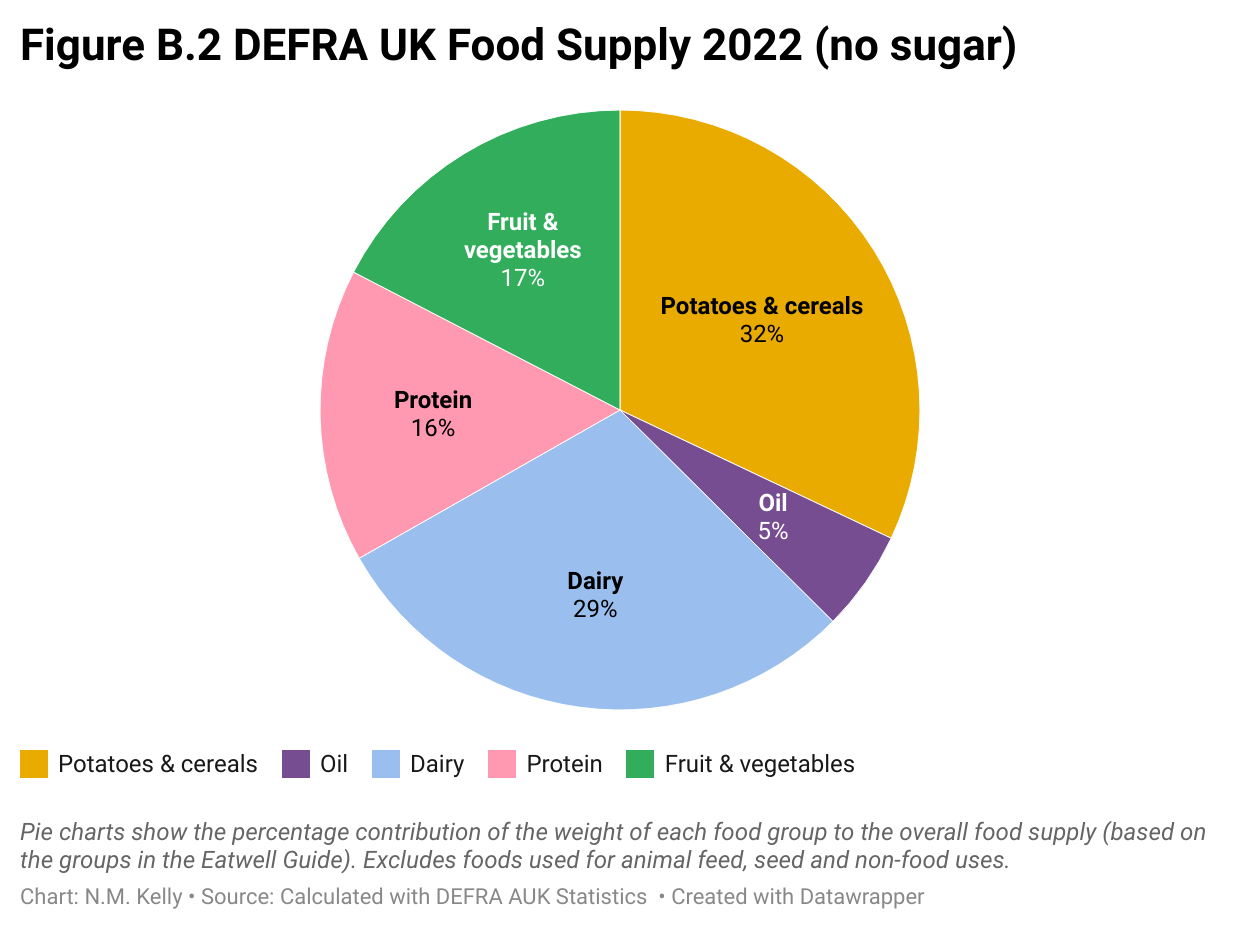


**Appendix D – UK Food Production, Imports and Exports from 2010-2022**

**Figure D1. UK Food Production from 2010-2022 divided by food group (million tonnes)**


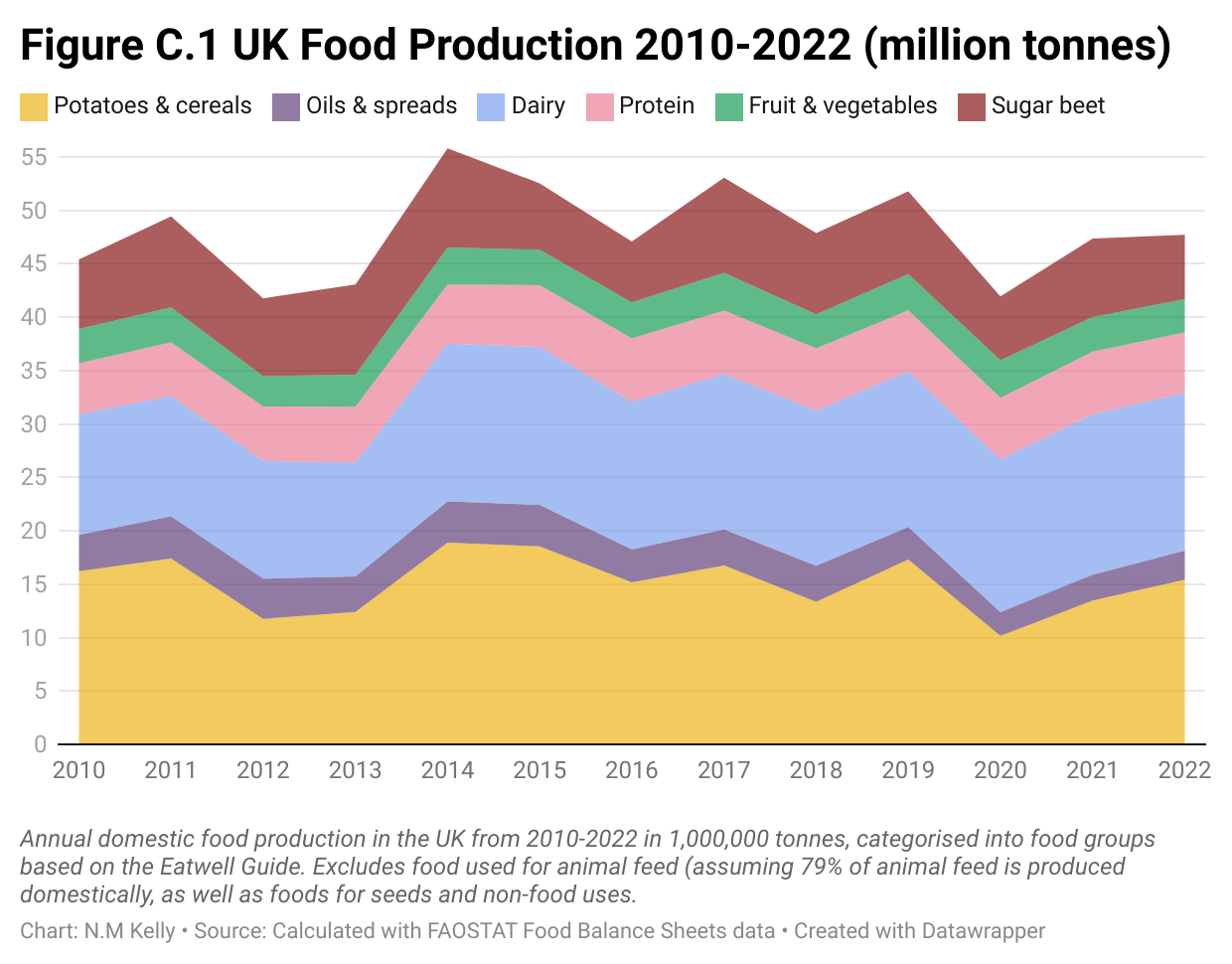


**Figure D2. UK Food Imports from 2010-2022 divided by food group (million tonnes)**

**
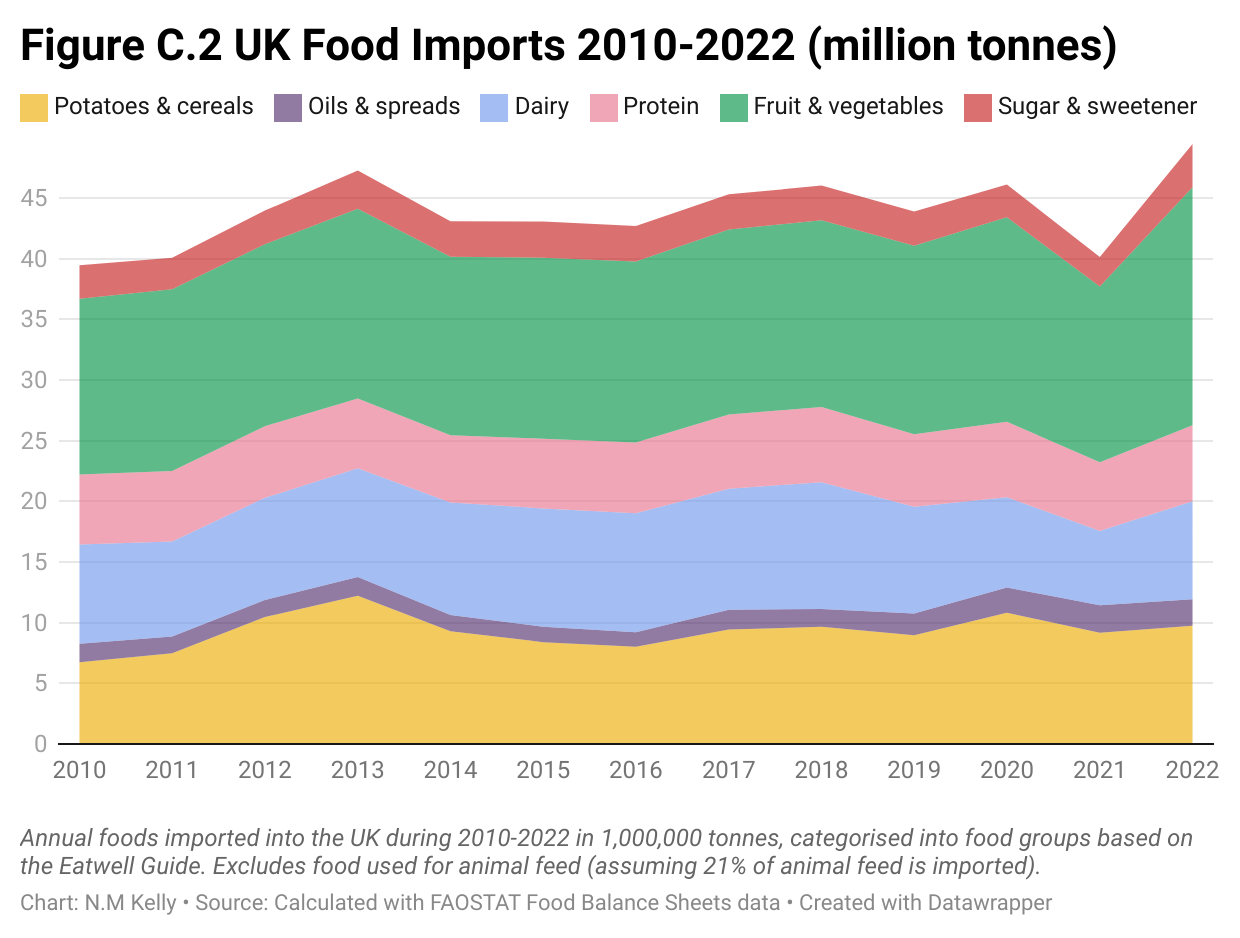
**

**Figure D3. UK Food Exports from 2010-2022 divided by food group (million tonnes)**


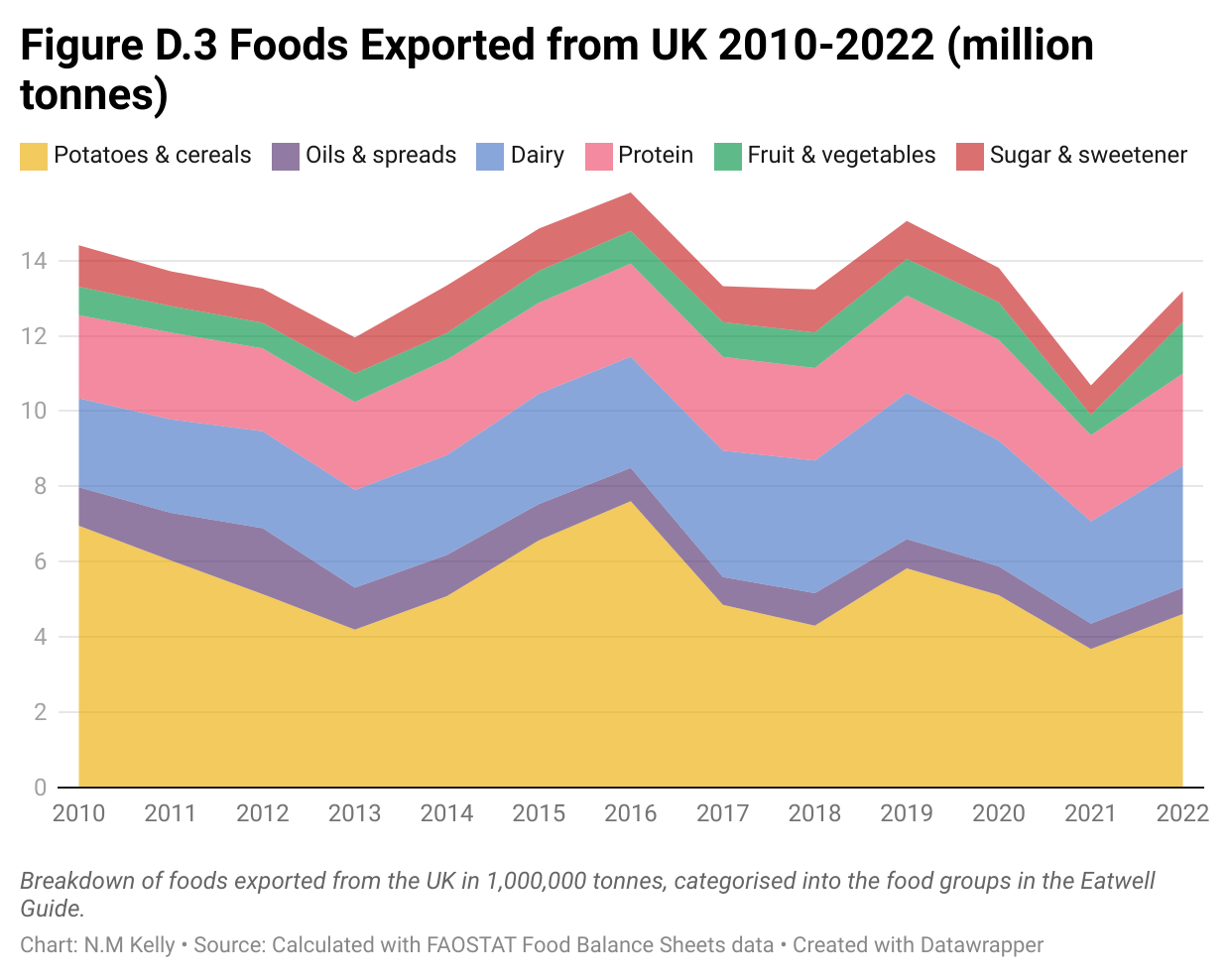


**Appendix E – Dietary Gap Assessment of Food and Feed supply for 2022**

**Figure E.1. Dietary Gap Assessment including Food and Feed Supply**

**
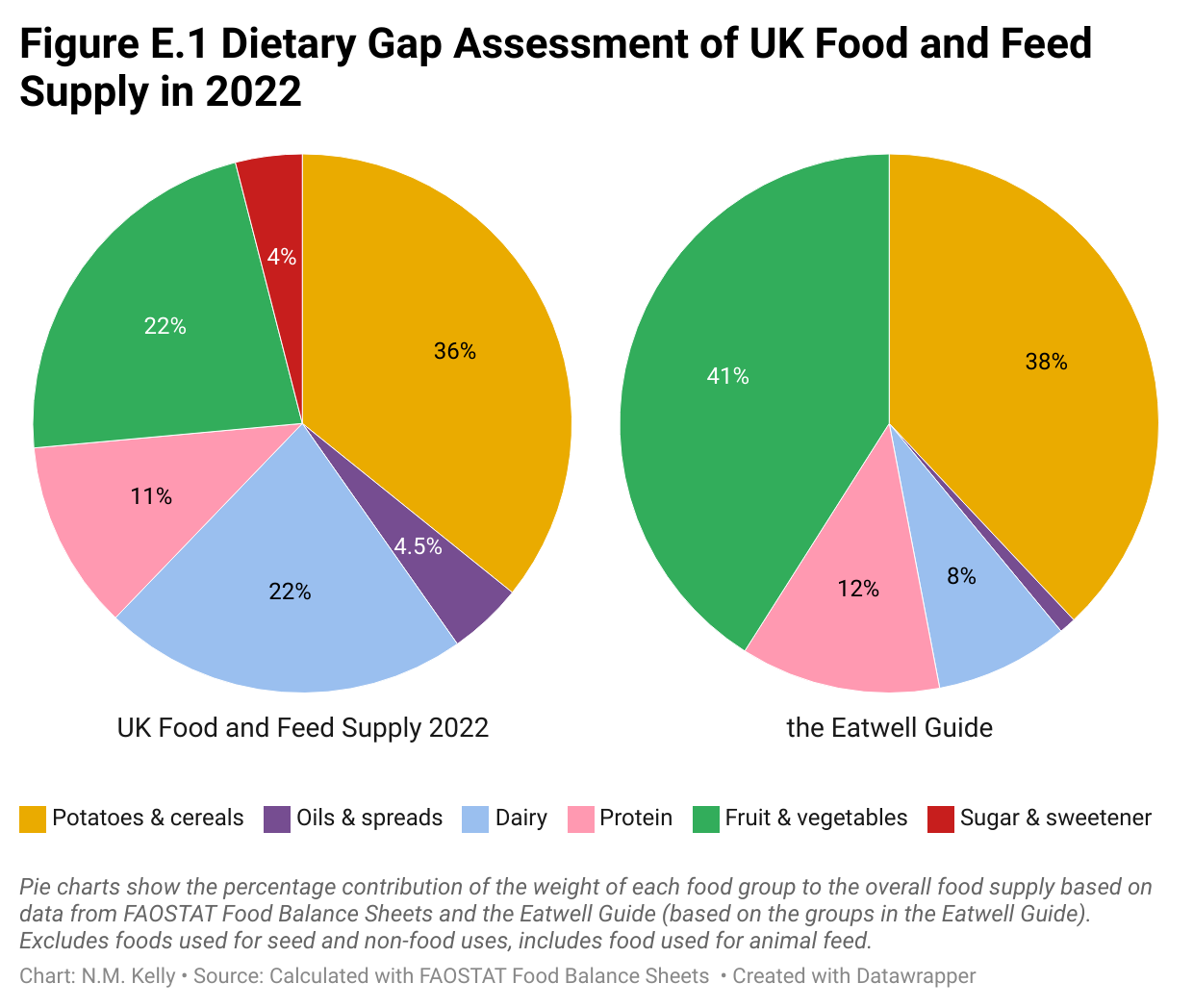
**

**Figure E.2. UK Food and Feed Supply breakdown by A) domestic production, B) imports, C) exports for 2022**
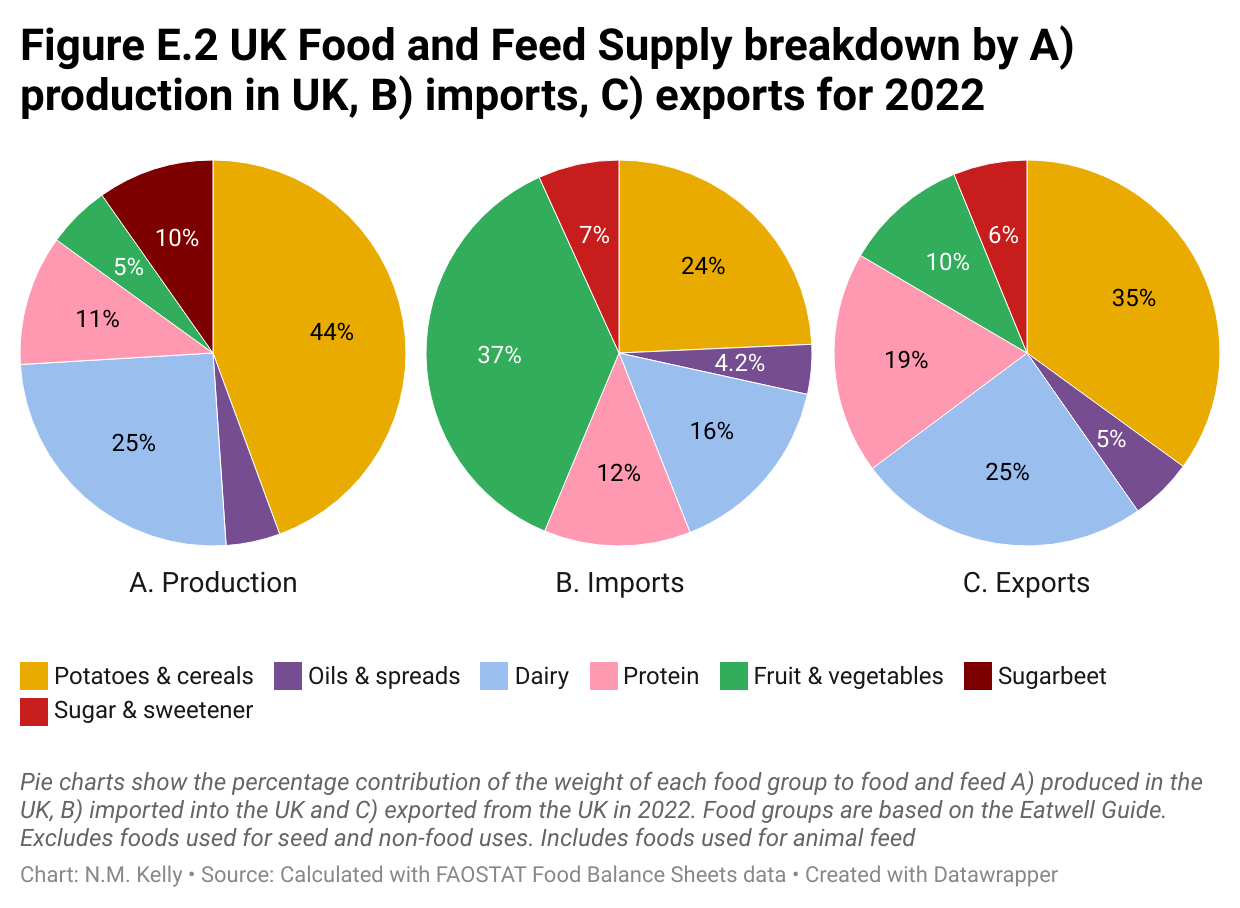

Supplement: Kelly et al. supplementary material 2 — Kelly et al. supplementary material [file S1368980025100633sup002.docx]
